# Epidemiology, risk factors and outcomes of prolonged mechanical ventilation with different cut-points in a PICU

**DOI:** 10.3389/fped.2023.1167595

**Published:** 2023-04-12

**Authors:** Tatchanapong Chongcharoenyanon, Rujipat Samransamruajkit, Jiratchaya Sophonphan

**Affiliations:** ^1^ Division of Pulmonology, Department of Pediatrics, King Chulalongkorn Memorial Hospital, Faculty of Medicine, Chulalongkorn University, Bangkok, Thailand; ^2^ Division of Pediatric Critical Care, Department of Pediatrics, King Chulalongkorn Memorial Hospital, Faculty of Medicine, Chulalongkorn University, Bangkok, Thailand; ^3^ The HIV Netherlands Australia Thailand Research Collaboration (HIV-NAT), Thai Red Cross AIDS Research Centre, Bangkok, Thailand

**Keywords:** prolong mechanical ventilation, children, pediatrics, definition, cut-point, long-term ventilation, incidence, tracheostomy

## Abstract

**Background:**

A consensus on the definition of prolonged mechanical ventilation (PMV) for children does not exist. There is still lack of published work presenting the epidemiology, risk factors and outcomes at different cut-points for PMV patients. These are important for planning the goals of treatment and counseling of the prognosis for patient families. We aimed to determine the incidence, baseline characteristics, risk factors and outcomes of PMV in pediatric patients at various cut-points (>14, >21 or >30days).

**Methods:**

A retrospective cohort study among children <18-years-old who were PMV > 14 days in the PICU of King Chulalongkorn Memorial Hospital was conducted. The primary outcomes were incidence of PMV with various cut-points. We stratified patients into three groups (Group 1; PMV > 14–21, Group 2; >21–30, Group 3; >30 days) for evaluating the baseline characteristics, risk factors, and outcomes of PMV (extubation success, tracheostomy status and death). Factors associated with PMV and deaths were analyzed using univariate and multivariate logistic regression.

**Results:**

From January 2018 to August 2022, 1,050 patients were screened. Of these, 114 patients were enrolled. The incidence of PMV > 14, >21 and >30 days were 10.9%, 7.3% and 5.0% respectively. Extubation success was significantly lower in Group 3 than in Groups 1 & 2 (15.4% vs. 62.2% & 56.0%, *P *< 0.001). Consequently, the tracheostomy rate (63.5% vs. 16.2% & 12.0%, *P *< 0.001), VAP rate (98.1% vs. 59.5% & 80.0%*, P *< 0.001), mortality rate by disease (34.6% vs. 5.4% & 20.0%*, P *= 0.003), median PICU LOS (50.5 vs. 22.0 & 28.0 days, *P *< 0.001) and median hospital LOS (124.5 vs. 55.0 & 62.0 days, *P *< 0.001) were also significantly higher for Group 3 compared with Groups 1 & 2. The factor associated with PMV > 30 days was VAP (aOR: 19.53, 95% CI: 2.38–160.34, *P* = 0.01). Factors associated with non-surviving patients were 3rd degree PEM (aOR: 5.14, 95% CI: 1.57–16.88, *P *= 0.01), PIM3 score ≥14 (aOR: 6.75, 95% CI: 2.26–20.15, *P *< 0.001) and muscle relaxant usage (aOR: 5.58, 95% CI: 1.65–18.86, *P *= 0.01).

**Conclusion:**

Extubation failure, tracheostomy rate, VAP rate, mortality rate by disease, PICU LOS and hospital LOS were significantly higher for PMV >30 days. Consequently, we suggest that a 30-day duration as a cut-point for PMV in PICUs might be more appropriate.

## Introduction

Medical advances, lower mortality and increased morbidity have increased the number of patients requiring prolonged mechanical ventilation (PMV) (1). In 2005, the National Association for Medical Direction of Respiratory Care (NAMDRC) defined PMV for adults as the need for ≥21 consecutive days of mechanical ventilation for ≥6 h/day of invasive and/or noninvasive methods of delivery. This cut-point was for most adult patients who were transferred to a long-term acute care (LTAC) hospital and had received ventilation for at least 21 days. The rate of PMV incidence in USA was estimated to be 3%–7% of mechanical ventilation (MV) patients (2–4). However, the PMV rate in China was surprisingly high, 36.1% of adults in ICUs (5).

Currently, a consensus of the definition of PMV for children does not exist. Therefore, published literature reports show a variability in the duration of PMV ranging from 2 to 30 days (6–11), a lack of standardization about the inclusion of noninvasive ventilation (NIV), and a lack of standardization about the inclusion of times when a child is ventilator-free during weaning (12). José Colleti Jr et al. reviewed the existing definitions of PMV in children and found the most frequent thresholds in duration cited were 7, 14, 21 and 30 days (11). However, existing evidence and our experience found that the majority of patients in PICUs can be extubated before 14 days and MV for more than 15 days is associated with extubation failure (6, 7, 13). Recently, Liu et al. reported the incidence of PMV > 14 days of a PICU in China was 33.2% (14). Three studies used a PMV cut-point >21 days showing an incidence of approximately 2.5%–9.0%. Chronic comorbidities were reported in 85% of the patients. Mortality ranged from 37.7% to 48.0% (1, 9–11). Ramírez et al. conducted a study of PMV > 30 days in thirty-three PICUs in Spain. The incidence where PMV > 30 days was 30% (15). The published risk factors associated with PMV were younger age, especially <1 year old, chronic comorbidities, prematurity, a higher PIM-3 score, inotropic or vasopressors usage, higher driving pressure and fluid overload (14, 16–21).

To the best of our knowledge, there is still a lack of published work presenting the epidemiology, risk factors and outcomes after different cut-points of PMV in children. This study aimed to determine the incidence of PMV in pediatric patients after different cut-points (>14, >21 or >30 days) and discover baseline characteristics, risk factors and outcomes of PMV in three groups (Group 1; >14–21 days, Group 2; >21–30 days, and Group 3; >30 days).

## Methods

### Study design and settings

This retrospective cohort study was conducted at King Chulalongkorn Memorial Hospital (KCMH), a tertiary university hospital in Bangkok, Thailand. It has a pediatric department of 300 beds and usually an average 6,500 outpatient visits, 1,000 inpatient and 40–50 PICU admissions per month. The study population included patients aged from 37 weeks postmenstrual age (PMA) to 18 years old admitted into the PICU during January 2018 to August 2022. Eligibility criteria for enrollment included (1) patients who were ventilated >14 consecutive days (short interruptions with <48 h of ventilation), (2) those on an invasive mechanical ventilator (*via* an endotracheal tube, nasotracheal tube or tracheostomy tube). Patients were excluded if they were already included in this study, were ventilated *via* NIV, were brain dead, premature children <37 weeks PMA at enrollment, or were home ventilator-dependent patients. The criteria of brain death were adopted from the Determination of Brain Death/Death by Neurologic Criteria: The World Brain Death Project (22).

This study was approved by the Institutional Review Board of the Faculty of Medicine, Chulalongkorn University (IRB No. 273/65) and informed consent requirements were waived. All data were kept confidential.

### Data collection and definition of variables

Data were extracted from medical record to case record forms, including age (months), gender, weight, underlying disease, main diagnosis related with the current PICU admission, underlying disease, preterm birth, nutritional status, cumulative fluid accumulation (FA) from Days 1–7 of MV, treatment information (maximum dynamic driving pressure, MV duration, respiratory complications during MV, minimum P/F ratio in the first 72 h, vasopressor or inotropes, maximum vasoactive-inotropic score (VIS) in the first 72 h, neuromuscular blocking agents and duration of neuromuscular blocking agents). Severity of illness scores such as pediatric index of mortality (PIM)-3 (23), pediatric logistic organ dysfunction (PELOD)-2 (24) scores were collected.

Pre-term was defined as patient born at a gestational age of <37 weeks. Underlying diseases were the condition of a patient prior to PICU admission. Nutritional status was categorized as either normal, 1st degree protein energy malnutrition (PEM), 2nd degree PEM or 3rd degree PEM (25, 26). Cumulative FA was calculated as the fluid inputs minus fluid outputs as a proportion of the admission (or preoperative) weight multiplied by 100. Fluids were expressed in liters and weights in kilograms. Fluid inputs included enteral and intravenous fluid administration. Fluid outputs included urine plus all other sources of fluid loss except insensible fluid loss (16, 27). Dynamic driving pressure (DP) was PIP minus PEEP. The dynamic DP overestimated the actual DP, but it could be used when patients are ventilated using modes that do not have a zero-flow state (17, 21). MV duration was defined as the time elapsed from intubation to extubation or successful disconnection from MV for tracheostomized patients (28, 29). The diagnosis of ventilator-associated pneumonia (VAP) was based on the CDC/NNIS definition related to age-specific criteria or qualified intensivist diagnosis by clinical assessment of clinical, laboratory, radiographic, and culture results (30). Hospital length of stay (LOS) was defined as the length of time elapsed between a patient's hospital admittance and discharge. PICU LOS was calculated as number of calendar days from the day of PICU admission (counted as 1 day) to the day of PICU discharge (31).

### Outcomes

The primary outcome was incidence of PMV by different cut-points (>14 days, >21 days or >30 days). The secondary outcomes were baseline characteristics, risk factors, PICU length of stay, hospital length of stay, respiratory complications (VAP, pneumomediastinum, pneumothorax, pulmonary hemorrhage and unplanned extubation). Outcomes of PMV include extubation success, tracheostomy with or without home ventilator-dependence, transfer back with MV to previous hospital and death. The patients were placed into one of three groups (Group 1; >14 to ≤21 days, Group 2; >21 and ≤30 days, Group 3; >30 days).

Indications and timing of tracheostomy were considered by the treating pediatric intensive care physician. Indications for tracheostomy were classified into three categories: upper airway obstruction, PMV and neurological impairment (such as cerebral palsy, encephalopathies with a Glasgow Coma Scale <8, central and peripheral neuromuscular disorders) (32, 33).

All outcomes were collected from the day ventilation was started until the patient was discharged. The patients who died after withdrawal therapy were also excluded because the MV duration might have been longer if treatment was continued.

### Statistical analyses

Demographic and clinical data were determined for each patient. Continuous variables are expressed as median (interquartile range: IQR) and percentage for categorical variables. Differences in continuous variables between the three groups were assessed using a Kruskal-Wallis test. A Wilcoxon rank sum test was used for comparisons between two groups. The categorical variables between groups were evaluated using *χ*^2^ or Fisher exact tests. Logistic regression (LR) was used to determine the factors associated PMV and death. Multivariate models were developed by adjusting for covariates with *P* < 0.1 in univariate models and stepwise backward LR to select a final model. All *P*-values reported are two-sided. Statistical significance was defined as *P* < 0.05. Stata version 15.1 (Stata Corp., College Station, Texas) was used for analysis.

## Results

### Patient demographic data and PMV incidence at various cut-points

**Figure 1:**
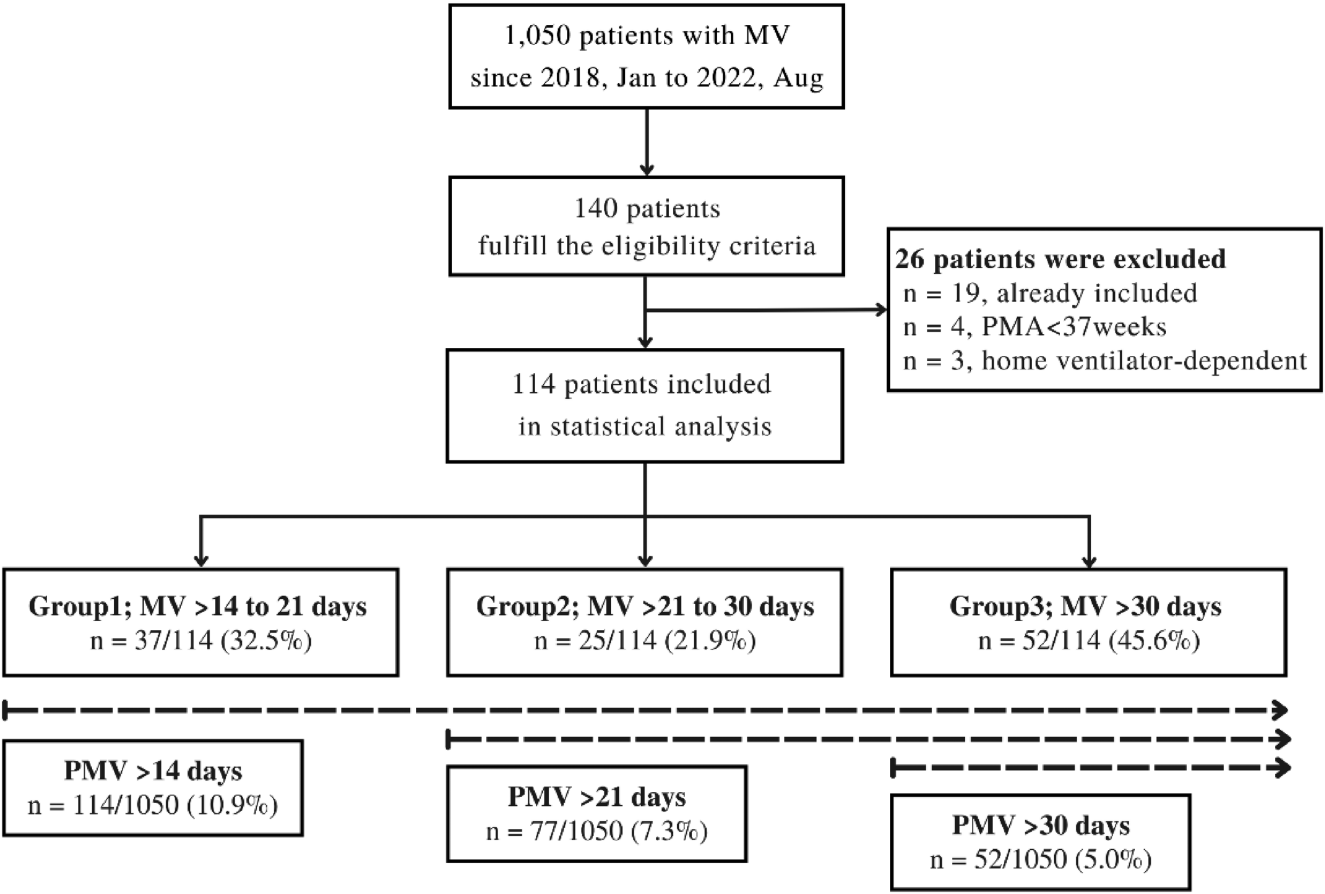
Flow diagram of study design. MV, mechanical ventilation; PMA, postmenstrual age; PMV, prolong mechanical ventilation.

From January 2018 to August 2022, 1,050 mechanically ventilated pediatric patients were screened. One hundred and forty patients fulfilled the eligibility criteria. Twenty-six patients were excluded. The most common reason for study exclusion is that patients were previous included in the study (*n* = 19/26). One hundred and fourteen patients were included in the statistical analysis. No patients were being mechanically ventilated through a tracheostomy tube. We stratified them into three groups, Group 1 (*n* = 37, 32.5%), Group 2 (*n* = 25, 21.9%) and Group 3 (*n* = 52, 45.6%). The incidence of PMV > 14 days was 10.9% (95% CI: 9.0–12.9), PMV > 21 days was 7.3% (95% CI: 5.8–9.1) and PMV > 30 days was 5.0% (95% CI: 3.7–6.4) (Figure 1).

The median age of all patients was 13 (IQR: 3–84) months. Females were 50.9% of all patients. The median MV duration was 26.5 days (IQR: 20.0–50.0). Most common main diagnoses were pulmonary disease, cardiac disease and neurological disease (58.8%, 15.8% and 10.5% respectively). Seventy-nine percent of the patients had underlying diseases. The most common underlying disease was cardiovascular disease which required significantly longer MV. A quarter of the patients were born prematurely (26.3%). Sixty percent of patients were malnourished, which was profoundly higher in Group 3 than Group 1 (71.2% vs. 48.7%, *P *< 0.05). The duration of MV was significantly longer for those with higher PIM-3 and PELOD2 scores. Baseline demographic data are shown in Table 1.

**Table 1:** Demographic and baseline data characteristics.

	Total (*n* = 114)	Group 1 (*n* = 37)	Group 2 (*n* = 25)	Group 3 (*n* = 52)	*P-*value
Age (months), median (IQR)	13 (3.0–84.0)	13 (5.0–48.0)	11 (4.0–34.0)	13 (3.0–115.0)	0.78
0–11 months old	54 (47.4%)	18 (48.7%)	13 (52.0%)	23 (44.2%)	
12–59 months old	27 (23.7%)	10 (27.0%)	6 (24.0%)	11 (21.2%)	
60–119 months old	12 (10.5%)	6 (16.2%)	0 (0)	6 (11.5%)	
120–215 months old	21 (18.4%)	3 (8.1%)	6 (24.0%)	12 (23.1%)	
Female sex, *n* (%)	58 (50.9)	21 (56.8)	10 (40.0)	27 (51.9)	0.42
Weight (kg), median (IQR)	8.2 (4.0–22.0)	7.3 (4.4–15.9)	8.5 (5.6–15.0)	9.1 (3.8–24.5)	0.92
MV duration (days), median (IQR)	26.5 (20.0–50.0)	17.0 (16.0–19.0)	25.0 (23.0–26.0)	54.5 (38.5–100.5)	<0.001
Main diagnosis, *n* (%)					0.44
Pulmonary disease	67 (58.8)	21 (56.8)	16 (64)	30 (57.7)	
Cardiac disease	18 (15.8)	4 (10.8)	3 (12)	11 (21.2)	
Neurological disease	12 (10.5)	3 (8.1)	2 (8)	7 (13.5)	
Post-surgery	7 (6.1)	5 (13.5)	1 (4)	1 (1.9)	
Shock	10 (8.8)	4 (10.8)	3 (12)	3 (5.8)	
Underlying disease, *n* (%)					0.84
No underlying disease	24 (21.1)	9 (24.3)	5 (20)	10 (19.2)	
Yes	90 (79)	28 (75.7)	20 (80)	42 (80.8)	
Cardiovascular disease	20 (17.5)	2 (5.4)*	6 (24)	12 (23.1)*	0.04
Pulmonary disease	17 (14.9)	6 (16.2)	2 (8)	9 (17.3)	0.59
Gastrointestinal disease	15 (13.2)	7 (18.9)	4 (16)	4 (7.7)	0.28
Oncological disease	8 (7)	2 (5.4)	1 (4)	5 (9.6)	0.71
Neuromuscular disease	7 (6.1)	2 (5.4)	1 (4)	4 (7.7)	0.80
Primary immune deficiency	4 (3.5)	1 (2.7)	2 (8)	1 (1.9)	0.43
Organ transplant	3 (2.6)	3 (8.1)	0 (0)	0 (0)	0.07
Others	16 (14)	5 (13.5)	4 (16)	7 (13.5)	0.95
Prematurity, *n* (%)	30 (26.3)	8 (21.6)	6 (24)	16 (30.8)	0.60
Malnutrition, *n* (%)					0.09
No	45 (39.5)	19 (51.4)	11 (44.0)	15 (28.9)	
Yes	69 (60.5)	18 (48.7)*	14 (56.0)	37 (71.2)*	
1st degree PEM	22 (19.3)	7 (18.9)	4 (16.0)	11 (21.2)	0.86
2nd degree PEM	16 (14.0)	4 (10.8)	4 (16.0)	8 (15.4)	0.79
3rd degree PEM	31 (27.2)	7 (18.9)	6 (24.0)	18 (34.6)	0.24
PIM-3 score (%), median (IQR)	6.9 (4.4–17.2)	5.5 (3.1–9.3)*	7.5 (5.7–22.6)*	7.6 (4.1–17.3)	0.04
PELOD-2 score, median (IQR)	5.0 (3.0–7.0)	4.0 (3.0–5.0)*	5.0 (4.0–6.0)	5.0 (4.0–7.0)*	0.04

MV, mechanical ventilation; PEM, protein energy malnutrition; PIM-3, pediatric index of mortality 3; PELOD-2, pediatric logistic organ dysfunction 2; Group 1, MV > 14–21 days; Group 2, MV > 21–30 days; Group 3, MV > 30 days; Differences in continuous and categorical variables between three groups using a Kruskal-Wallis test; Differences in continuous variables between two groups using a Wilcoxon rank sum test; Differences in categorical variables used *χ*^2^ test or Fisher exact test.

* Statistically significant for pairwise comparison (*P* < 0.05).

### Treatment information

The median of the minimum P/F ratio in the first 72 h was 169 (IQR: 118–268). It was significantly lower in Group 3 than Group 1 (150, IQR: 104–206 vs. 222, IQR: 139–283, *P* = 0.04).

More than half of the patients (58.8%) were using inotropic or vasopressor drugs with a median maximum VIS in the first 72 h of 10.0 (IQR: 0–20.0). Muscle relaxant was used for 49.1% of the patients with a median duration of 4.5 days (IQR: 2.5–11.5). The median maximum DP was 16 cm H_2_O (IQR: 13–20). The median maximum cumulative FA was 9.5% (IQR: 5.6–17.7) with a median of 6 days after MV (IQR: 3–7). The median cumulative FA from Day 1 to Day 7 is shown in Table 2. However, there was no statistical difference in these parameters between groups.

**Table 2:** Treatment information.

	Total (*n* = 114)	Group 1 (*n* = 37)	Group 2 (*n* = 25)	Group 3 (*n* = 52)	*P-*value
Inotropic or vasopressor usage, *n* (%)	67 (58.8)	19 (51.4)	14 (56.0)	34 (65.4)	0.39
Maximum VIS in the first 72 h, median (IQR)	10.0 (0–20.0)	5.0 (0–15.0)	5.0 (0–25.0)	10.0 (0–27.9)	0.31
Minimum P/F ratio in the first 72 h, median (IQR)	169 (118–268)	222 (139–283)*	182 (122–272)	150 (104–206)*	0.04
Maximum DP (cmH_2_O), median (IQR)	16 (13–20)	15 (12–20)	15 (14–18)	17 (14–20)	0.18
Muscle relaxant usage, *n* (%)	56 (49.1)	15 (40.5)	12 (48.0)	29 (55.8)	0.36
Duration of muscle relaxant (days), median (IQR)	4.5 (2.5–11.5)	3 (2.0–7.0)	3 (2.5–11.5)	5 (3.0–18.0)	0.13
Cumulative FA Day 1 (%), median (IQR)	2.7 (0.5–4.4)	2.8 (0.6–5.1)	2.9 (0.9–4.4)	2.4 (0–4.2)	0.55
Cumulative FA Day 2 (%), median (IQR)	4.1 (0.5–6.8)	2.5 (0.8–8.2)	4.7 (−0.1–6.4)	4.1 (0.8–6.3)	0.84
Cumulative FA Day 3 (%), median (IQR)	4.8 (0.6–8.7)	5.5 (1.5–10.3)	5.1 (0.4–7.4)	4 (−0.2–8.1)	0.50
Cumulative FA Day 4 (%), median (IQR)	5.2 (0.6–9.9)	5.7 (0.8–10.1)	5.3 (1.2–9.3)	4.1 (0.2–10.4)	0.80
Cumulative FA Day 5 (%), median (IQR)	5.3 (0.3–12.1)	6.6 (0.4–12.1)	5.2 (−0.1–10.8)	5.2 (0.3–12.3)	0.90
Cumulative FA Day 6 (%), median (IQR)	7.1 (0.2–13.8)	4.9 (−1.0–13.8)	7.5 (4.0–12.7)	7.1 (−1.0–18)	0.82
Cumulative FA Day 7 (%), median (IQR)	6.5 (1.0–16.3)	5.5 (−1.2–15.6)	7.9 (4.7–14.7)	6.6 (−0.5–22.6)	0.54
Maximum cumulative FA (%), median (IQR)	9.5 (5.6–17.7)	8.9 (5.3–16.4)	9.6 (6.1–16.2)	9.7 (4.5–23.1)	0.79
Days of maximum cumulative FA, median (IQR)	6 (3–7)	5 (3–7)	6.5 (4–7)	6 (3–7)	0.56

PMV, prolonged mechanical ventilation; VIS, vasoactive-inotropic score; DP, driving pressure; FA, fluid accumulation; Group 1, MV > 14–21 days; Group 2, MV > 21–30 days; Group 3, MV > 30 days; Differences in continuous and categorical variables between three groups using a Kruskal-Wallis test; Differences in continuous variables between two groups using a Wilcoxon rank sum test; Differences in categorical variables used *χ*^2^ test or Fisher exact test.

* Statistically significant for pairwise comparisons (*P* < 0.05).

### Clinical outcomes after PMV

The extubation success rate was at 45/114 (39.5%) and it was significantly higher for Group 1 and Group 2 than for Group 3 (62.2% and 56.0% vs. 15.4%, *P* < 0.001). Consequently, Group 3 statistically had the highest tracheostomy rate (*n* = 33/52, 63.5%), VAP rate (*n* = 51/52, 98.1%), PICU LOS (median 50.5 days, IQR: 39.5–76.0) and hospital LOS (median 124.5 days, IQR: 74.5–206). Overall, the median mortality rate was 33/114 (29.0%) and 25/114 (21.9%) after exclusion of patients withdrawn from therapy. It is noteworthy that the highest mortality was in Group 3 (*n* = 18/52, 34.6%). Almost all patients of Group 1 were tracheostomized with indications of upper airway obstruction (*n* = 5/6, 83.3%). In contrast, within Group 3, the most common indication was PMV (*n* = 18/33, 54.6%). The median days intubated before tracheostomy was significantly higher for Group 3 (39 days, IQR: 22–59, *P *< 0.02) (Table 3).

**Table 3:** Outcomes after PMV.

Parameter	Total (*n* = 114)	Group 1 (*n* = 37)	Group 2 (*n* = 25)	Group 3 (*n* = 52)	*P-*value
Extubation success, *n* (%)	45 (39.5)	23 (62.2)*	14 (56.0)^#^	8 (15.4)*,^#^	<0.001
Tracheostomy, *n* (%)	42 (36.8)	6 (16.2)*	3 (12.0)^#^	33 (63.5)*,^#^	<0.001
Tracheostomy without ventilator-dependent, *n* (%)	28 (24.6)	5 (13.5)*	2 (8.0)^#^	21 (40.4)*,^#^	0.001
Tracheostomy with ventilator-dependent, *n* (%)	14 (12.3)	1 (2.7)*	1 (4.0)	12 (23.1)*	0.006
Indication for tracheostomy, *n* (%)					
Upper airway obstruction	16/42 (38.1)	5/6 (83.3)*	1/3 (33.3)	10/33 (30.3)*	0.04
PMV	19/42 (45.2)	1/6 (16.7)	0/3 (0)	18/33 (54.6)	0.08
Neuromuscular disease	7/42 (16.7)	0/6 (0)	2/3 (66.7)	5/33 (15.2)	0.06
Days intubated before tracheostomy, median (IQR)	30 (18–54)	18 (17–18)*,^#^	26 (19–42)*	39 (22–59)^#^	0.02
Death, *n* (%)	33 (29.0)	8 (21.6)	7 (28.0)	18 (34.6)	0.41
Death by disease, *n* (%)	25 (21.9)	2 (5.4)*	5 (20.0)	18 (34.6)*	0.03
Death by withdrawal therapy, *n* (%)	8 (7.0)	6 (16.2)*	2 (8.0)	0 (0)*	0.005
Transfer back with MV, *n* (%)	6 (5.3)	0 (0)	1 (4.0)	5 (9.6)	0.10
Respiratory complication during MV, *n* (%)					<0.001
No complication	17 (14.9)	12 (32.4)	4 (16.0)	1 (1.9)	
Yes	97 (85.1)	25 (67.6)	21 (84.0)	51 (98.1)	
VAP	93 (81.6)	22 (59.5)*	20 (80.0)^#^	51 (98.1)*,^#^	<0.001
Pneumothorax	14 (12.3)	4 (10.8)	3 (12.0)	7 (13.5)	0.94
Pulmonary hemorrhage	8 (7.0)	2 (5.4)	4 (16.0)	2 (3.9)	0.17
Unplanned extubation	2 (1.8)	0	1 (4.0)	1 (1.9)	0.50
Pneumomediastinum	0	0	0	0	
PICU LOS (days), median (IQR)	35.0	22.0	28.0	50.5	<0.001^¥^
	(23.0–54.0)	(18.0–28.0)	(25.0–35.0)	(39.5–76.0)	
Hospital LOS (days), median (IQR)	79.0	55.0	62.0	124.5	<0.001
	(52.0–141.0)	(43.0–87.0)*	(48.0–102.0)^#^	(74.5–206.0)*,^#^	

PMV, prolonged mechanical ventilation; MV, mechanical ventilation; VAP, ventilator-associated pneumonia; LOS, length of stay; Group 1, MV > 14–21 days; Group 2, MV > 21–30 days; Group 3, MV > 30 days; Differences in continuous and categorical variables between three groups using a Kruskal-Wallis test; Differences in continuous variables between two groups using a Wilcoxon rank sum test; Differences in categorical variables used a *χ*^2^ test or Fisher exact test.

* Statistical significant for pairwise comparison (*P* < 0.05).

# Statistical significant for pairwise comparison (*P* < 0.05).

¥ All pairwise comparisons are statistical significantly.

### PMV > 14–30 days vs. PMV > 30 days

**Figure 2:**
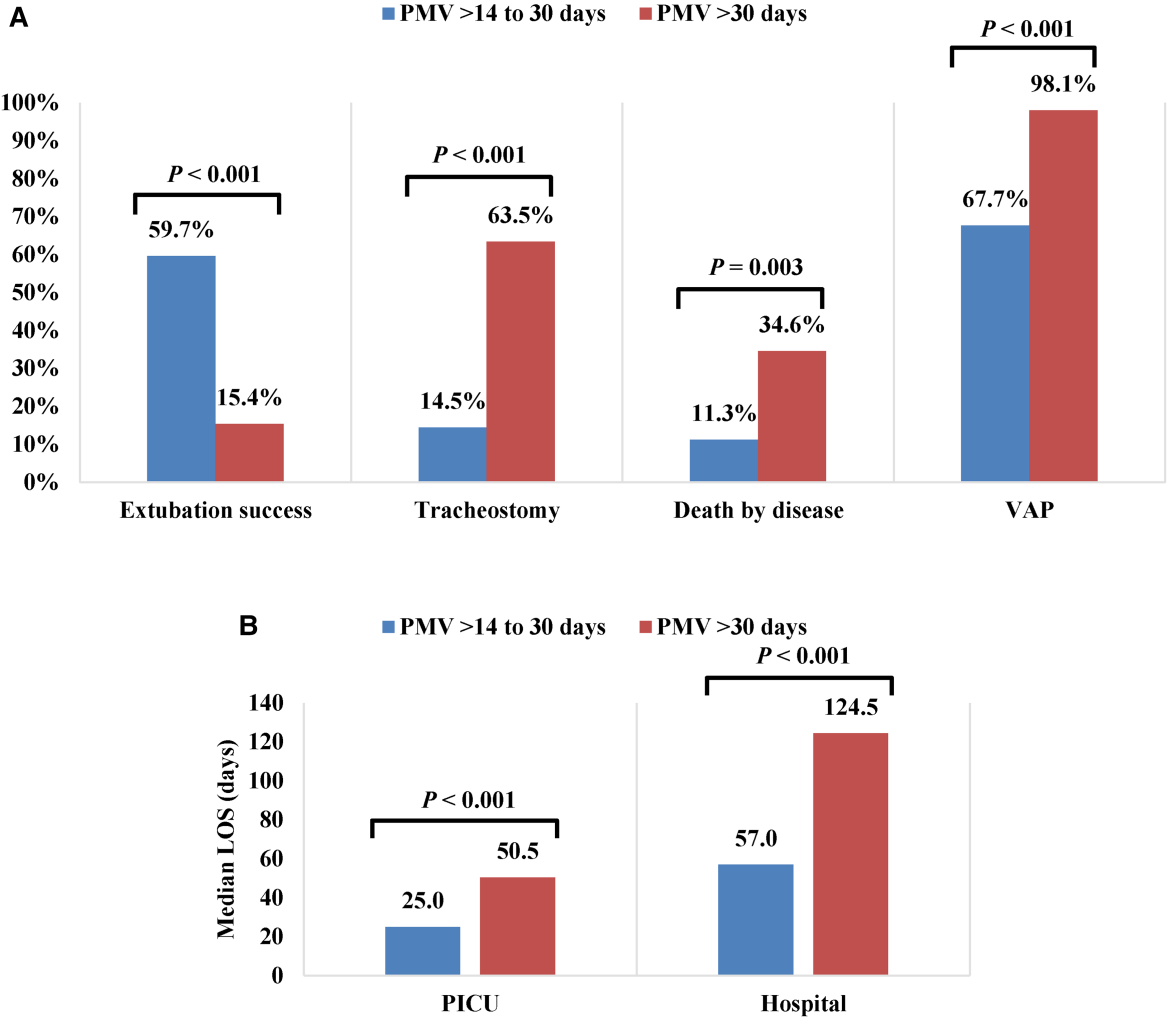
Outcomes of patients with PMV > 14 to 30 days and PMV > 30 days (**A**) Percentage of extubation success, tracheotomy, death by disease and VAP (**B**) Median PICU and hospital LOS (days). PMV, Prolonged Mechanical Ventilation; VAP, Ventilator-Associated Pneumonia; LOS, Length Of Stay.

According to data for outcomes after PMV, we decided to re-categorize study participants to underscore the obvious differences between two groups: PMV > 14–30 days (combining Group 1 and Group 2) and PMV > 30 days (Group 3) (Supplementary material Tables S1–S3). In the PMV > 30-days group, the extubation success rate (15.4% vs. 59.7%, *P *< 0.001) and median minimum P/F ratio in the first 72 h were profoundly lower than for PMV > 14–30 days [150 (104–206) vs. 195.5 (132–275), *P* = 0.02]. The tracheostomy rate (63.5% vs. 14.5%, *P* < 0.001), PMV as an indication of tracheostomy rate (54.6% vs. 11.1%, *P* < 0.001), mortality rate after excluded withdrawal therapy (34.6% vs. 11.3%, *P* = 0.003), VAP rate (98.1% vs. 67.7%, *P* < 0.001), median duration of muscle relaxant use [5 (3–18) vs. 3 (2–8) days, *P* = 0.05], median PICU LOS [50.5 (39.5–76.0) vs. 25.0 (20.0–35.0) days, *P *< 0.001] and median hospital LOS [124.5 (74.5–206.0) vs. 57.0 (43.0–89.0) days, *P* < 0.001] were higher in the PMV > 30-days group (Figure 2).

**Figure 3:**
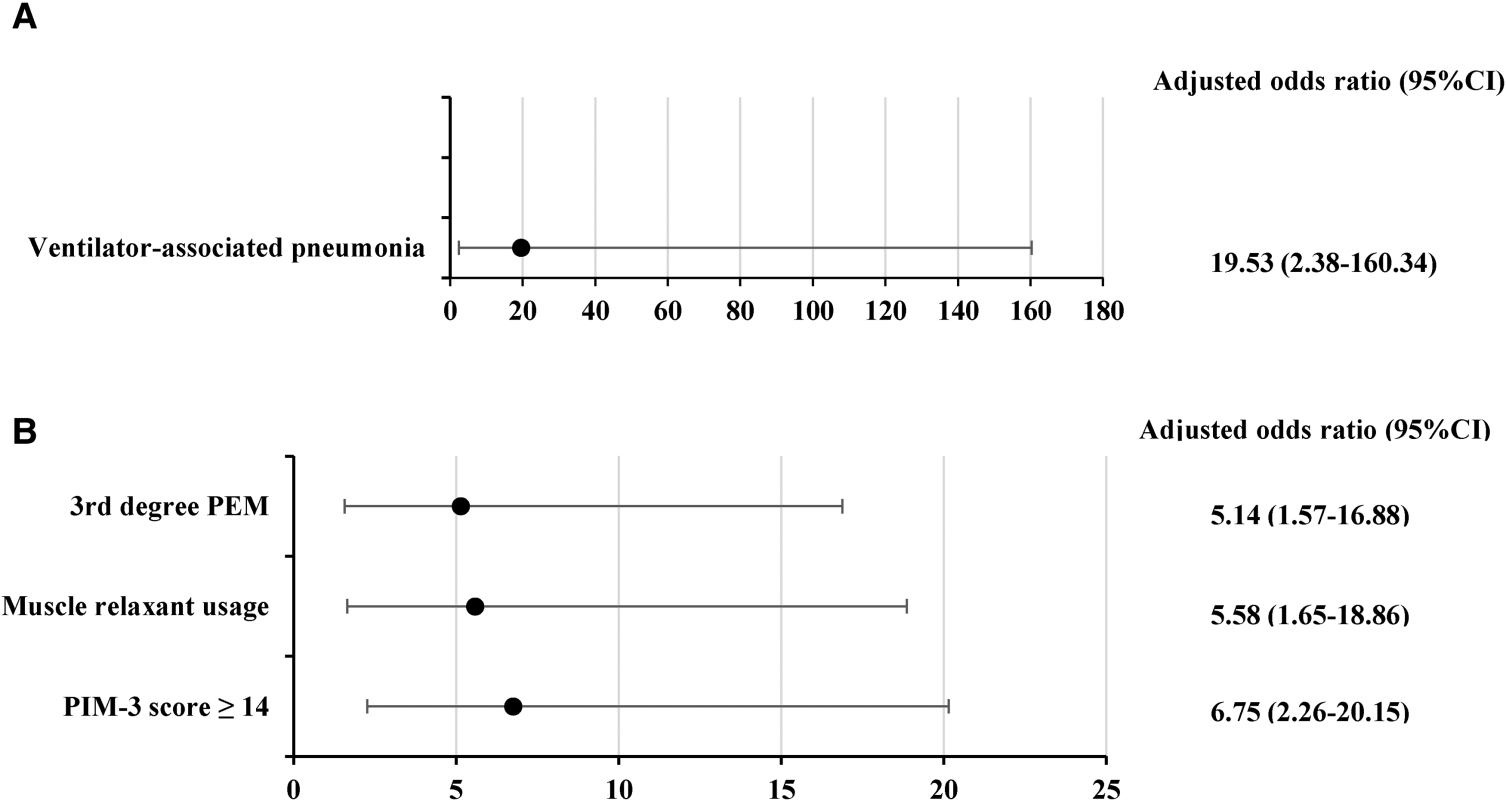
Factors associated with **(A)** PMV > 30 days **(B)** deaths; Data were analyzed using a multivariate logistic regression model. PEM, protein energy malnutrition; PIM-3, pediatric index of mortality 3.

Univariate and multivariate analyses through binary logistic regression were performed to compare the patients in the PMV > 14–30 days and PMV > 30 days groups (Supplementary material Table S4). Multivariable analysis showed that only VAP was associated with PMV > 30 days (aOR: 19.53, 95% CI: 2.38–160.34, *P =* 0.01) (Figure 3A).

### Survivors vs. deaths

A comparison between survivors (*n* = 81/114, 71.1%) and deaths (*n* = 33/114, 28.9%) is shown in Supplementary material Tables S5–S7. Deaths had higher rates of cardiac disease as a main diagnosis (27.3% vs. 11.1%, *P* = 0.03), cardiovascular diseases (30.3% vs. 12.4%, *P* = 0.02) and gastrointestinal diseases (24.2% vs. 8.6%, *P* = 0.03) as underlying diseases, 3rd degree PEM (54.6% vs. 16.1%, *P* < 0.001), inotropic or vasopressor usage (78.8% vs. 50.6%, *P* = 0.006), muscle relaxant usage (75.8% vs. 38.3%, *P* < 0.001), median maximum VIS in the first 72 h [20 (7–47) vs. 2.8 (0–15), *P* < 0.001], median duration of muscle relaxant use [10 (5–15) vs. 3 (2–5) days, *P* < 0.001], median maximum DP [19 (16–22) vs. 15 (12–18) cm H_2_O, *P* < 0.001], median PIM-3 score [17.3 (8.5–22.4) vs. 5.7 (3.3–9.6), *P* < 0.001] and median of PELOD-2 score [5 (3–6) vs. 6 (3–7), *P* = 0.03]. Risk factors associated with death patients were 3rd degree PEM (aOR: 5.14, 95% CI: 1.57–16.88, *P* = 0.01), PIM3 score ≥14 (aOR: 6.75, 95% CI: 2.26–20.15, *P* < 0.001) and muscle relaxant use (aOR: 5.58, 95% CI: 1.65–18.86, *P =* 0.01) (Figure 3B).

## Discussion

The current study reports the incidence of PMV > 14, >21 and >30 days was 10.9%, 7.3% and 5.0%, respectively. We decided to not include the NIV patients because they can be managed outside the PICU. This might be the reason that our incidence of PMV > 14 days was lower than a previous study reported from China PICU (33.2%) (14). The incidence of PMV > 21 days was close to that reported from an Argentinian PICU (9%), but higher than a study in Brazilian PICUs (2.5%) and an adult ICU (3–7%). However, our mortality rate for PMV > 21 days was 32.5% (*n* = 25/77), which was lower than for Brazilian PICUs (48.0%), Argentinian PICUs (43.0%), in a Taiwan database (37.7%) and an adult ICU (42.4%) (1, 4, 9, 10, 34). This indicates high mortality rates for PMV > 21 days. Furthermore, a study in 33 Spanish PICUs reported 30% of patients required PMV > 30 days, whereas our institution reported a much lower level. Differences in patient characteristics might explain this.

In our study, we observed that 26.3% of patients were born prematurely and 60% of patients suffered from malnutrition. These factors could have been potential contributors to the requirement for PMV in some cases. Premature birth can lead to underdeveloped lungs and respiratory muscles, which can result in respiratory failure and the need for mechanical ventilation. Furthermore, complications like bronchopulmonary dysplasia may prolong the necessity for mechanical ventilation in premature infants. Meanwhile, malnutrition can weaken respiratory muscles, thereby increasing the likelihood of respiratory failure and the subsequent need for mechanical ventilation. Additionally, malnutrition may cause other complications that can contribute to PMV, such as infections or organ failure. Teka et al. concluded that malnutrition in PICU was a frequent cause of morbidity that was linked with a higher requirement for mechanical ventilation (35).

Several studies have reported baseline characteristics, risk factors and outcomes using arbitrary cut-points for PMV (11). To identify the PMV effect on clinical outcomes, we illustrate this by using different cut-point durations for PMV. In 2005, Fontela et al. first showed that MV ≥ 15 days was associated with a low extubation success rate (4.5%), whereas our study revealed a much higher success rate (39.5%) (13). This finding suggests that knowledge advancements about weaning and support care can improve the extubation success rate over time. Furthermore, we also found that extubation success was not different between PMV > 14–21 days (62.2%) and PMV > 21–30 days (56.0%), but it was significantly lower for PMV > 30 days (15.4%). Damuth et al. revealed a 29.0% pooled mortality at hospital discharge in PMV > 14 days for adults, which is similar to our results (36). We underscore that the mortality rate after excluded withdrawal therapy was significantly higher for PMV > 30 days compared with PMV > 14–30 days (34.6% vs. 11.3%, *P* = 0.003). Factors associated with mortality were inotropic or vasopressor usage, PIM-3 score ≥14% and muscle relaxant usage. Additionally, our analysis revealed that individuals who died had a significantly higher incidence of cardiac disease as a main diagnosis (27.3% vs. 11.1%, *P* = 0.03), as well as higher rates of cardiovascular (30.3% vs. 12.4%, *P* = 0.02) and gastrointestinal diseases (24.2% vs. 8.6%, *P* = 0.03) as underlying diseases. They also had a higher median maximum DP [19 (16–22) vs. 15 (12–18) cm H_2_O, *P* < 0.001] compared to the survivors. Our findings suggest that patients with cardiac disease are more vulnerable to changes in fluid and electrolyte balance, and poor cardiac function can worsen respiratory complications such as atelectasis or pulmonary edema, which can ultimately lead to death.

Although it was recommended for adult patients that tracheostomy should be performed within 1–2 weeks of ventilation, no consensus criteria of time to tracheostomy exists for children (32, 37). Some studies reported that early tracheostomy (within 14 days of ventilation) could reduce MV duration and hospital LOS (38, 39). From another point of view, caregivers of children with tracheostomy tubes have high caregiver workloads and financial burden (40, 41). Our institution is highly concern about these problems. Therefore, we tend to delay tracheostomy tube for indications of PMV. The tracheostomy rate due to PMV was significantly higher for PMV > 30 days compared with PMV for 14–30 days (*n* = 18/52, 34.6% vs. *n* = 1/62, 1.6%, *P* = 0.02). The results of our study could be applied for family counseling regarding the prognosis for PMV in children. We found that if we used a cut point of PMV > 30 days, we could identify the highest percentage of children who eventually required tracheostomy. Thus, we suggest that use of a 30-day duration as a cut-point for PMV in PICUs might be more appropriate. The mortality rate and other morbidity data that we found in our study such as the VAP rate, PICU LOS and hospital LOS were also the highest for PMV > 30 days.

High DP, more than 15 cm H_2_O, in acute hypoxemic respiratory failure children was independently associated with longer MV duration and PICU LOS, but not in the mortality rate. There is currently no study of DP in PMV. The median maximum DP in our study was 16 cm H_2_O (IQR: 13–20) and DP tended to be greater when comparing PMV > 30 days with PMV > 14–30 days [17 (14–20) vs. 15 (13–18), *P* = 0.09] which supports previous studies (17, 21). Although deaths had a significantly higher median maximum DP than survivors [19 (16–22) vs. 15 (12–18) cm H_2_O, *P* < 0.001], it was not an independent predictor of mortality after multivariate analysis (even with a cutoff of 17 cm H_2_O). Several studies reported an association between fluid accumulation and MV duration, PICU LOS and mortality rate (18–20). Recently, Gelbart et al. reported moderate FA up to 10% in MV critically ill children was not associated with harm in contrast with extremely greater than 20% which was associated with higher morbidity and mortality (16). Our institution is also greatly concerned about this issue. Consequently, the median cumulative FA from Day 1 to 7 was less than 10% and a median maximal FA was 9.5%. Therefore, we did not find any significance of FA among the three groups of PMV patients. There were no significant differences between survivors and deaths. Our particular findings highlight that a FA of less than 10% may not be associated with morbidity and mortality in PMV patients.

To the best of our knowledge, this is the first study that evaluates baseline characteristics, risk factors and outcomes using various definitions instead of arbitrary cut-points for duration of PMV in a PICU. However, our study has some limitations. First, it was conducted in a single tertiary care setting where the incidence of PMV is higher and mortality rate is lower than in other settings. This may limit the generalizability of the findings. Nevertheless, we would like to propose the idea of conducting further studies that use various cut-points instead of single cut-points for duration of PMV. Second, it was a retrospective study that mainly relied on medical chart review. However, we can extract more than 95% of the data due to the use of electronic records. Third, most of our patients had an underlying disease that might influence the results. Fourth, we only included invasively mechanically ventilated children. In the future, a multicenter study using different cut-points for PMV in children seems warranted.

## Data Availability

The original contributions presented in the study are included in the article/**Supplementary Materials**, further inquiries can be directed to the corresponding author.

## References

[1] Pai S-C Kung P-T Chou W-Y Kuo T Tsai W-C. Survival and medical utilization of children and adolescents with prolonged ventilator-dependent and associated factors. PLoS One. (2017) 12(6):e0179274. 10.1371/journal.pone.0179274 28628663 PMC5476277

[2] Hassenpflug M Scheinhorn DJ Chao DC Palma C. Post-ICU mechanical ventilation: treatment of 2,369 patients over 16 years at a regional weaning center. Chest. (2004) 126(4):750S. 10.1378/chest.126.4_MeetingAbstracts.750S-a 9187189

[3] Scheinhorn DJ Chao DC Hassenpflug MS Gracey DR. Post-ICU weaning from mechanical ventilation: the role of long-term facilities. Chest. (2001) 120(6, Supplement):482S–4S. 10.1378/chest.120.6_suppl.482S 11742970

[4] MacIntyre NR Epstein SK Carson S Scheinhorn D Christopher K Muldoon S. Management of patients requiring prolonged mechanical ventilation: report of a NAMDRC consensus conference. Chest. (2005) 128(6):3937–54. 10.1378/chest.128.6.3937 16354866

[5] Li J Zhan QY Wang C. Survey of prolonged mechanical ventilation in intensive care units in mainland China. Respir Care. (2016) 61(9):1224–31. 10.4187/respcare.04295 27460102

[6] Wakeham MK Kuhn EM Lee KJ McCrory MC Scanlon MC. Use of tracheostomy in the PICU among patients requiring prolonged mechanical ventilation. Intensive Care Med. (2014) 40(6):863–70. 10.1007/s00134-014-3298-4 24789618

[7] Payen V Jouvet P Lacroix J Ducruet T Gauvin F. Risk factors associated with increased length of mechanical ventilation in children. Pediatr Crit Care Med. (2012) 13(2):152–7. 10.1097/PCC.0b013e3182257a24 21760567

[8] Polito A Patorno E Costello JM Salvin JW Emani SM Rajagopal S Perioperative factors associated with prolonged mechanical ventilation after complex congenital heart surgery*. Pediatr Crit Care Med. (2011) 12(3):e122–6. 10.1097/PCC.0b013e3181e912bd 20625334

[9] Monteverde E Fernández A Poterala R Vidal N Siaba Serrate A Castelani P Characterization of pediatric patients receiving prolonged mechanical ventilation. Pediatr Crit Care Med. (2011) 12(6):e287–91. 10.1097/PCC.0b013e3182191c0b 21499185

[10] Traiber C Piva JP Fritsher CC Garcia PCR Lago PM Trotta EA Profile and consequences of children requiring prolonged mechanical ventilation in three Brazilian pediatric intensive care units. Pediatr Crit Care Med. (2009) 10(3):375–80. 10.1097/PCC.0b013e3181a3225d 19325502

[11] Colleti JJ Azevedo RT de Oliveira Caino FR de Araujo OR. Prolonged mechanical ventilation in children: review of the definition. Pediatr Crit Care Med. (2021) 22(11):e588–93. 10.1097/PCC.0000000000002773 34028375

[12] Sauthier M Rose L Jouvet P. Pediatric prolonged mechanical ventilation: considerations for definitional criteria. Respir Care. (2017) 62(1):49. 10.4187/respcare.04881 27879381

[13] Fontela PS Piva JP Garcia PC Bered PL Zilles K. Risk factors for extubation failure in mechanically ventilated pediatric patients. Pediatr Crit Care Med. (2005) 6(2):166–70. 10.1097/01.PCC.0000154922.65189.48 15730603

[14] Liu Y Wang Q Hu J Zhou F Liu C Li J Characteristics and risk factors of children requiring prolonged mechanical ventilation vs. non-prolonged mechanical ventilation in the PICU: a prospective single-center study. Front Pediatr. (2022) 10:830075. 10.3389/fped.2022.830075 35211431 PMC8861196

[15] Ramírez JB Cid JL-H Alapont VM. Prevalencia de la ventilación mecánica en las unidades de cuidados intensivos pediátricos en españa. Anales de Pediatría. (2004) 61(6):533–41. 10.1016/S1695-4033(04)78440-4 15574254

[16] Gelbart B Serpa Neto A Stephens D Thompson J Bellomo R Butt W Fluid accumulation in mechanically ventilated, critically ill children: retrospective cohort study of prevalence and outcome. Pediatr Crit Care Med. (2022) 23(12):990–8. 10.1097/PCC.0000000000003047 36454001

[17] van Schelven P Koopman AA Burgerhof JGM Markhorst DG Blokpoel RGT Kneyber MCJ. Driving pressure is associated with outcome in pediatric acute respiratory failure. Pediatr Crit Care Med. (2022) 23(3):e136–44. 10.1097/PCC.0000000000002848 34669679 PMC8897270

[18] Kong X Zhu Y Zhu X. Association between early fluid overload and mortality in critically-ill mechanically ventilated children: a single-center retrospective cohort study. BMC Pediatr. (2021) 21(1):474. 10.1186/s12887-021-02949-w 34702226 PMC8549157

[19] Lopes CLS Piva JP. Fluid overload in children undergoing mechanical ventilation. Rev Bras Ter Intensiva. (2017) 29(3):346–53. 10.5935/0103-507X.20170045 28977099 PMC5632978

[20] van Mourik N Metske HA Hofstra JJ Binnekade JM Geerts BF Schultz MJ Cumulative fluid balance predicts mortality and increases time on mechanical ventilation in ARDS patients: an observational cohort study. PLoS One. (2019) 14(10):e0224563-e. 10.1371/journal.pone.0224563 31665179 PMC6821102

[21] Rauf A Sachdev A Venkataraman ST Dinand V. Dynamic airway driving pressure and outcomes in children with acute hypoxemic respiratory failure. Respir Care. (2021) 66(3):403–9. 10.4187/respcare.08024 33024000

[22] Greer DM Shemie SD Lewis A Torrance S Varelas P Goldenberg FD Determination of brain death/death by neurologic criteria: the world brain death project. JAMA. (2020) 324(11):1078–97. 10.1001/jama.2020.11586 32761206

[23] Straney L Clements A Parslow RC Pearson G Shann F Alexander J Paediatric index of mortality 3: an updated model for predicting mortality in pediatric intensive care*. Pediatr Crit Care Med. (2013) 14(7):673–81. 10.1097/PCC.0b013e31829760cf 23863821

[24] Leteurtre S Duhamel A Salleron J Grandbastien B Lacroix J Leclerc F PELOD-2: an update of the PEdiatric logistic organ dysfunction score. Crit Care Med. (2013) 41(7):1761–73. 10.1097/CCM.0b013e31828a2bbd 23685639

[25] Bhattacharyya AK. Protein-energy malnutrition (kwashiorkor-marasmus syndrome): terminology, classification and evolution. World Rev Nutr Diet. (1986) 47:80–133. 10.1159/000412332 3088855

[26] Waterlow JC. Classification and definition of protein-energy malnutrition. Monogr Ser World Health Organ. (1976) (62):530–55. PMID: 824854. 824854

[27] Abulebda K Cvijanovich NZ Thomas NJ Allen GL Anas N Bigham MT Post-ICU admission fluid balance and pediatric septic shock outcomes: a risk-stratified analysis. Crit Care Med. (2014) 42(2):397–403. 10.1097/CCM.0b013e3182a64607 24145842 PMC3947064

[28] Gamberini L Tonetti T Spadaro S Zani G Mazzoli CA Capozzi C Factors influencing liberation from mechanical ventilation in coronavirus disease 2019: multicenter observational study in fifteen Italian ICUs. J Intensive Care. (2020) 8(1):80. 10.1186/s40560-020-00499-4 33078076 PMC7558552

[29] Contentin L Ehrmann S Giraudeau B. Heterogeneity in the definition of mechanical ventilation duration and ventilator-free days. Am J Respir Crit Care Med. (2014) 189(8):998–1002. 10.1164/rccm.201308-1499LE 24735035

[30] Srinivasan R Asselin J Gildengorin G Wiener-Kronish J Flori HR. A prospective study of ventilator-associated pneumonia in children. Pediatrics. (2009) 123(4):1108–15. 10.1542/peds.2008-1211 19336369

[31] Williams TA Ho KM Dobb GJ Finn JC Knuiman M Webb SAR. Effect of length of stay in intensive care unit on hospital and long-term mortality of critically ill adult patients. Br J Anaesth. (2010) 104(4):459–64. 10.1093/bja/aeq025 20185517

[32] Watters KF. Tracheostomy in infants and children. Respir Care. (2017) 62(6):799. 10.4187/respcare.05366 28546379

[33] Can FK Anıl AB Anıl M Gümüşsoy M Çitlenbik H Kandoğan T The outcomes of children with tracheostomy in a tertiary care pediatric intensive care unit in Turkey. Turk Pediatri Ars. (2018) 53(3):177–84. 10.5152/TurkPediatriArs.2018.6586 30459517 PMC6239071

[34] Hill AD Fowler RA Burns KE Rose L Pinto RL Scales DC. Long-term outcomes and health care utilization after prolonged mechanical ventilation. Ann Am Thorac Soc. (2017) 14(3):355–62. 10.1513/AnnalsATS.201610-792OC 28033033

[35] Teka SG Kebede RA Sherman C. The prevalence of malnutrition during admission to the pediatric intensive care unit, a retrospective cross-sectional study at tikur anbessa specialized hospital, Addis Ababa, Ethiopia. Pan Afr Med J. (2022) 41:77. 10.11604/pamj.2022.41.77.31284 35382053 PMC8956830

[36] Damuth E Mitchell JA Bartock JL Roberts BW Trzeciak S. Long-term survival of critically ill patients treated with prolonged mechanical ventilation: a systematic review and meta-analysis. Lancet Respir Med. (2015) 3(7):544–53. 10.1016/S2213-2600(15)00150-2 26003390

[37] Andriolo BN Andriolo RB Saconato H Atallah ÁN Valente O. Early versus late tracheostomy for critically ill patients. Cochrane Database Syst Rev. (2015 1. 10.1002/14651858.CD010959.pub2 PMC6517297 25581416

[38] de Araujo OR Azevedo RT de Oliveira FRC Junior JC. Tracheostomy practices in children on mechanical ventilation: a systematic review and meta-analysis. J Pediatr. (2022) 98(2):126–35. 10.1016/j.jped.2021.07.004 PMC9432186 34509427

[39] Chorath K Hoang A Rajasekaran K Moreira A. Association of early vs late tracheostomy placement with pneumonia and ventilator days in critically ill patients: a meta-analysis. JAMA Otolaryngol Head Neck Surg. (2021) 147(5):450–9. 10.1001/jamaoto.2021.0025 33704354 PMC7953336

[40] Baddour K Mady LJ Schwarzbach HL Sabik LM Thomas TH McCoy JL Exploring caregiver burden and financial toxicity in caregivers of tracheostomy-dependent children. Int J Pediatr Otorhinolaryngol. (2021) 145:110713. 10.1016/j.ijporl.2021.110713 33882339

[41] Hartnick CJ Bissell C Parsons SK. The impact of pediatric tracheotomy on parental caregiver burden and health Status. Arch Otolaryngol Head Neck Surg. (2003) 129(10):1065–9. 10.1001/archotol.129.10.1065 14568788

