# Corrigendum: Epidemiology, risk factors and outcomes of prolonged mechanical ventilation with different cut-points in a PICU

**DOI:** 10.3389/fped.2023.1237299

**Published:** 2023-06-27

**Authors:** Tatchanapong Chongcharoenyanon, Rujipat Samransamruajkit, Jiratchaya Sophonphan

**Affiliations:** ^1^ Division of Pulmonology, Department of Pediatrics, King Chulalongkorn Memorial Hospital, Faculty of Medicine, Chulalongkorn University, Bangkok, Thailand; ^2^ Division of Pediatric Critical Care, Department of Pediatrics, King Chulalongkorn Memorial Hospital, Faculty of Medicine, Chulalongkorn University, Bangkok, Thailand; ^3^ The HIV Netherlands Australia Thailand Research Collaboration (HIV-NAT), Thai Red Cross AIDS Research Centre, Bangkok, Thailand

**Keywords:** prolong mechanical ventilation, children, pediatrics, definition, cut-point, long-term ventilation, incidence, tracheostomy

A Corrigendum on Epidemiology, risk factors and outcomes of prolonged mechanical ventilation with different cut-points in a PICU By Chongcharoenyanon T, Samransamruajkit R and Sophonphan J. (2023) Front. Pediatr. 11:1167595. doi: 10.3389/fped.2023.1167595

**Error in Author List**

In the published article, there was an error in the author list, and authors Jiratchaya Sophonphan and Rujipat Samransamruajkit are in the incorrect order. As a result the order of affiliations have also changed. The corrected author list and affiliations appears below.

Tatchanapong Chongcharoenyanon^1^, Rujipat Samransamruajkit^2^, Jiratchaya Sophonphan^3^

^1^Division of Pulmonology, Department of Pediatrics, King Chulalongkorn Memorial Hospital, Faculty of Medicine, Chulalongkorn University, Bangkok, Thailand, ^2^Division of Pediatric Critical Care, Department of Pediatrics, King Chulalongkorn Memorial Hospital, Faculty of Medicine, Chulalongkorn University, Bangkok, Thailand, ^3^The HIV Netherlands Australia Thailand Research Collaboration (HIV-NAT), Thai Red Cross AIDS Research Centre, Bangkok, Thailand

The authors apologize for this error and state that this does not change the scientific conclusions of the article in any way. The original article has been updated.

**Error in Correspondence**

In the published article, there was an error in the correspondence, and author Tatchanapong Chongcharoenyanon was stated as the corresponding author. The correct corresponding author appears below.

Rujipat Samransamruajkit, rujipatrs@gmail.com

The authors apologize for this error and state that this does not change the scientific conclusions of the article in any way. The original article has been updated.

