# Supplementary material for: Epidemiology, risk factors and outcomes of prolonged mechanical ventilation with different cut-points in a PICU
Source: Front Pediatr. 2023 Apr 12;11:1167595. doi: 10.3389/fped.2023.1167595 (PMC10130509; doi:10.3389/fped.2023.1167595)
Supplement: Supplementary file 1 [file Table1.docx]

Supplementary Material

**Supplementary Table 1 |** Demographic and baseline data characteristics between patients with PMV >14 to 30 days and PMV >30 days.

|  | **PMV >14 to 30 days**  **(n= 62)** | **PMV > 30 days**  **(n= 52)** | ***P-*value** |
| --- | --- | --- | --- |
| **Age (months), median (IQR)** | 12 (4-48) | 13 (3-115) | 0.61 |
| 0 to 11 months old | 31 (50%) | 23 (44.2%) |  |
| 12 to 59 months old | 16 (25.8%) | 11 (21.2%) |  |
| 60 to 119 months old | 6 (9.7%) | 6 (11.5%) |  |
| 120 to 215 months old | 9 (14.5%) | 12 (23.1%) |  |
| **Female sex, n (%)** | 31 (50.0) | 27 (51.9) | 0.84 |
| **Weight (kg), median (IQR)** | 7.5 (4.5-15.9) | 9.1 (3.8-24.5) | 0.75 |
| **MV duration (days), median (IQR)** | 21 (17.0-24.0) | 54.5 (38.5-100.5) | <0.001 |
| **Main diagnosis, n (%)** |  |  | 0.18 |
| Pulmonary disease | 37 (59.7) | 30 (57.7) |  |
| Cardiac disease | 7 (11.3) | 11 (21.2) |  |
| Neurological disease | 5 (8.1) | 7 (13.5) |  |
| Post-surgery | 7 (11.3) | 3 (5.8) |  |
| Shock | 6 (9.7) | 1 (1.9) |  |
| **Underlying disease, n (%)** |  |  | 0.66 |
| No underlying disease | 14 (22.6) | 10 (19.2) |  |
| Yes | 48 (77.4) | 42 (80.8) |  |
| Cardiovascular disease | 8 (12.9) | 12 (23.1) | 0.16 |
| Pulmonary disease | 8 (12.9) | 9 (17.3) | 0.60 |
| Gastrointestinal disease | 11 (17.7) | 4 (7.7) | 0.16 |
| Neuromuscular disease | 3 (4.8) | 4 (7.7) | 0.70 |
| Oncological disease | 3 (4.8) | 5 (9.6) | 0.47 |
| Primary immune deficiency | 3 (4.8) | 1 (1.9) | 0.62 |
| Organ transplant | 3 (4.8) | 0 (0) | 0.25 |
| Others | 9 (14.5) | 7 (13.5) | 0.87 |
| **Prematurity, n (%)** | 14 (22.6) | 16 (30.8) | 0.32 |
| **Malnutrition, n (%)** |  |  | 0.04 |
| No | 30 (48.4) | 15 (28.9) |  |
| Yes | 32 (51.6) | 37 (71.2) |  |
| 1^st^ degree PEM | 11 (17.7) | 11 (21.2) | 0.65 |
| 2^nd^ degree PEM | 8 (12.9) | 8 (15.4) | 0.70 |
| 3^rd^ degree PEM | 13 (21) | 18 (34.6) | 0.14 |
| **PIM-3 score (%), median (IQR)** | 6.5 (4.7-16.1) | 7.6 (4.1-17.3) | 0.45 |
| **PELOD-2 score, median (IQR)** | 5 (3-6) | 5 (4-7) | 0.02 |

MV, Mechanical Ventilation; PEM, Protein Energy Malnutrition; PIM-3, Pediatric Index of Mortality 3; PELOD-2, Pediatric Logistic Organ Dysfunction 2; Differences in continuous variables between two groups using a Wilcoxon rank sum test; Differences in categorical variables used Chi-square test or Fisher exact test

**Supplementary Table 2 |** Treatment information between patients with PMV >14 to 30 days and PMV >30 days.

|  | **PMV >14 to 30 days**  **(n= 62)** | **PMV > 30 days**  **(n= 52)** | ***P-*value** |
| --- | --- | --- | --- |
| **Inotropic or vasopressor usage, n (%)** | 33 (53.2) | 34 (65.4) | 0.19 |
| **Maximum VIS in the first 72 hours, median (IQR)** | 5.0 (0-20.0) | 10.0 (0-27.9) | 0.21 |
| **Minimum P/F ratio in the first 72 hours, median (IQR)** | 195.5 (132-275) | 150 (104-206) | 0.02 |
| **Maximum DP (cmH2O), median (IQR)** | 15 (13-18) | 17 (14-20) | 0.09 |
| **Muscle relaxant usage, n (%)** | 27 (43.6) | 29 (55.8) | 0.19 |
| **Duration of muscle relaxant (days), median (IQR)** | 3 (2-8) | 5 (3-18) | 0.05 |
| **Cumulative FA Day1 (%), median (IQR)** | 2.9 (0.6-4.5) | 2.4 (0-4.2) | 0.34 |
| **Cumulative FA Day2 (%), median (IQR)** | 4.2 (0.2-7.4) | 4.1 (0.8-6.3) | 0.56 |
| **Cumulative FA Day3 (%), median (IQR)** | 5.2 (1.1-8.9) | 4 (-0.2-8.1) | 0.43 |
| **Cumulative FA Day4 (%), median (IQR)** | 5.6 (0.8-9.6) | 4.1 (0.2-10.4) | 0.57 |
| **Cumulative FA Day5 (%), median (IQR)** | 5.6 (0.2-12.1) | 5.2 (0.3-12.3) | 0.82 |
| **Cumulative FA Day6 (%), median (IQR)** | 7 (0.8-13.8) | 7.1 (-1-18) | 0.94 |
| **Cumulative FA Day7 (%), median (IQR)** | 6.5 (1.8-15.6) | 6.6 (-0.5-22.6) | 0.96 |
| **Maximum cumulative FA (%), median (IQR)** | 9.3 (5.8-16.4) | 9.7 (4.5-23.1) | 0.59 |
| **Days of maximum cumulative FA, median (IQR)** | 6 (3-7) | 6 (3-7) | 0.85 |

PMV, Prolonged Mechanical Ventilation; VIS, Vasoactive-Inotropic Score; DP, Driving Pressure; FA, Fluid Accumulation; Differences in continuous variables between two groups using a Wilcoxon rank sum test; Differences in categorical variables were used Chi-square test or Fisher exact test.

**Supplementary Table 3 |** Outcome after PMV between patients with PMV >14 to 30 days and PMV >30 days.

|  | **PMV >14 to 30 days**  **(n= 62)** | **PMV > 30 days**  **(n= 52)** | ***P-*value** |
| --- | --- | --- | --- |
| **Extubation success, n (%)** | 37 (59.7) | 8 (15.4) | <0.001 |
| **Tracheostomy, n (%)** | 9 (14.5) | 33 (63.5) | <0.001 |
| **Tracheostomy without ventilator-dependent, n (%)** | 7 (11.3) | 21 (40.4) | <0.001 |
| **Tracheostomy with ventilator-dependent, n (%)** | 2 (3.2) | 12 (23.1) | 0.001 |
| **Indication for tracheostomy, n (%)** |  |  |  |
| Upper airway obstruction | 6/9 (66.7) | 10/33 (30.3) | 0.06 |
| PMV | 1/9 (11.1) | 18/33 (54.6) | 0.02 |
| Neuromuscular disease | 2/9 (22.2) | 5/33 (15.2) | 0.47 |
| **Days intubated before tracheostomy, median (IQR)** | 18 (18-20) | 39 (22-59) | 0.01 |
| **Death, n (%)** | 15 (24.2) | 18 (34.6) | 0.22 |
| Death by disease, n (%) | 7 (11.3) | 18 (34.6) | 0.003 |
| Death by withdrawal therapy, n (%) | 8 (12.9) | 0 (0) | 0.008 |
| **Transfer back with MV, n (%)** | 1 (1.6) | 5 (9.6) | 0.09 |
| **Respiratory complication during MV, n (%)** |  |  | <0.001 |
| No complication | 16 (25.8) | 1 (1.9) |  |
| Yes | 46 (74.2) | 51 (98.1) |  |
| VAP | 42 (67.7) | 51 (98.1) | <0.001 |
| Pneumothorax | 7 (11.3) | 7 (13.5) | 0.73 |
| Pulmonary hemorrhage | 6 (9.7) | 2 (3.9) | 0.29 |
| Unplanned extubation | 1 (1.6) | 1 (1.9) | 0.90 |
| Pneumomediastinum | 0 | 0 |  |
| **PICU LOS (days), median (IQR)** | 25.0 (20.0-35.0) | 50.5 (39.5-76.0) | <0.001 |
| **Hospital LOS (days), median (IQR)** | 57.0 (43.0-89.0) | 124.5 (74.5-206.0) | <0.001 |

PMV, Prolonged Mechanical Ventilation; MV, Mechanical Ventilation; VAP, Ventilator-Associated Pneumonia; LOS, Length Of Stay; Differences in continuous variables between two groups using a Wilcoxon rank sum test; Differences in categorical variables were used Chi-square test or Fisher exact test.

**Supplementary Table 4 |** Logistic regression models assessing the variables associated with PMV >30 days.

|  | **Univariate analysis** | | **Multivariate analysis** | |
| --- | --- | --- | --- | --- |
|  | **OR (95% CI)** | ***P-*value** | **aOR (95% CI)** | ***P-*value** |
| **Age < 12 months** | 0.79 (0.38-1.66) | 0.54 |  |  |
| **Female** | 1.08 (0.52-2.26) | 0.84 |  |  |
| **Main diagnosis** |  |  |  |  |
| Pulmonary disease | 0.92 (0.44-1.95) | 0.83 |  |  |
| Cardiac disease | 2.11 (0.75-5.91) | 0.16 |  |  |
| Neurological disease | 1.77 (0.53-5.96) | 0.35 |  |  |
| Shock | 0.48 (0.12-1.96) | 0.31 |  |  |
| **Underlying disease** | 1.23 (0.49-3.05) | 0.66 |  |  |
| Pulmonary disease | 1.41 (0.5-3.97) | 0.51 |  |  |
| Cardiovascular disease | 2.03 (0.76-5.42) | 0.16 |  |  |
| Gastrointestinal disease | 0.39 (0.12-1.3) | 0.12 |  |  |
| Neuromuscular disease | 1.64 (0.35-7.68) | 0.53 |  |  |
| Oncological disease | 2.09 (0.48-9.21) | 0.33 |  |  |
| Primary immune deficiency | 0.39 (0.04-3.82) | 0.42 |  |  |
| **Prematurity** | 1.52 (0.66-3.52) | 0.32 |  |  |
| **Respiratory complication** |  |  |  |  |
| VAP | 24.29 (3.13-188.55) | <0.001 | 19.53 (2.38-160.34) | 0.01 |
| Pneumothorax | 1.22 (0.4-3.74) | 0.73 |  |  |
| Pulmonary hemorrhage | 0.37 (0.07-1.93) | 0.24 |  |  |
| **Malnutrition (1^st^-3^rd^ degree PEM)** | 2.31 (1.06-5.04) | 0.04 | 1.94 (0.79-4.75) | 0.15 |
| **Inotropic or vasopressor usage** | 1.66 (0.78-3.54) | 0.19 |  |  |
| **Maximum VIS in the first 72 ≥ 6** | 1.94 (0.92-4.11) | 0.08 | 2.04 (0.82-5.08) | 0.13 |
| **PIM-3 score ≥ 7** | 2.02 (0.95-4.27) | 0.07 | 1.08 (0.42-2.76) | 0.87 |
| **PELOD-2 score ≥ 5** | 1.9 (0.87-4.16) | 0.11 |  |  |
| **Minimum P/F ratio in the first 72 hour < 170** | 2.37 (1.11-5.04) | 0.03 | 1.26 (0.51-3.09) | 0.62 |
| **Maximum DP ≥ 15 cmH_2_O** | 2.17 (0.97-4.85) | 0.06 | 1.1 (0.41-2.97) | 0.85 |
| **Muscle relaxant usage** | 1.63 (0.78-3.43) | 0.20 |  |  |

VAP, Ventilation Associated Pneumonia; PEM, Protein Energy Malnutrition; VIS, Vasoactive-Inotropic Score; PIM-3, Pediatric Index of Mortality 3; PELOD-2, Pediatric Logistic Organ Dysfunction 2; DP, Driving Pressure; OR: Odds ratio, aOR : Adjusted odds ratio , Univariate and multivariate were performed by logistic regression model. Multivariate were developed by covariate with *P* < 0.1 from univariate.

**Supplementary Table 5 |** Demographic and baseline data characteristics between survivors and deaths.

|  | **Survivor**  **(N=81)** | **Deaths (N=33)** | ***P-*value** |
| --- | --- | --- | --- |
| **Age (months), median (IQR)** | 15 (4-92) | 11 (3-67) | 0.32 |
| 0 to 11 months old | 37 (45.7%) | 17 (51.5%) |  |
| 12 to 59 months old | 20 (24.7%) | 7 (21.2%) |  |
| 60 to 119 months old | 9 (11.1%) | 3 (9.1%) |  |
| 120 to 215 months old | 15 (18.5%) | 6 (18.2%) |  |
| **Female sex, n (%)** | 39 (48.2) | 19 (57.6) | 0.36 |
| **Weight (kg), median (IQR)** | 8.6 (4.1-23) | 6.5 (3.8-20) | 0.43 |
| **MV duration (days), median (IQR)** | 26 (20-46) | 33 (22-53) | 0.57 |
| **Main diagnosis, n (%)** |  |  |  |
| Pulmonary disease | 49 (60.5) | 18 (54.6) | 0.56 |
| Cardiac disease | 9 (11.1) | 9 (27.3) | 0.03 |
| Neurological disease | 9 (11.1) | 3 (9.1) | 0.75 |
| Post-surgery | 7 (8.6) | 0 (0) | 0.11 |
| Shock | 7 (8.6) | 3 (9.1) | 0.94 |
| **Underlying disease, n (%)** |  |  | 0.14 |
| No underlying disease | 20 (24.7) | 4 (12.1) |  |
| Yes | 61 (75.3) | 29 (87.9) |  |
| Pulmonary disease | 15 (18.5) | 2 (6.1) | 0.15 |
| Cardiovascular disease | 10 (12.4) | 10 (30.3) | 0.02 |
| Gastrointestinal disease | 7 (8.6) | 8 (24.2) | 0.03 |
| Neuromuscular disease | 6 (7.4) | 1 (3) | 0.67 |
| Oncological disease | 6 (7.4) | 2 (6.1) | 0.80 |
| Primary immune deficiency | 1 (1.2) | 3 (9.1) | 0.07 |
| Organ transplant | 3 (3.7) | 0 (0) | 0.56 |
| Others | 13 (16.1) | 3 (9.1) | 0.39 |
| **Prematurity, n (%)** | 22 (27.2) | 8 (24.2) | 0.75 |
| **Malnutrition, n (%)** |  |  | 0.39 |
| No | 34 (42) | 11 (33.3) |  |
| Yes | 47 (58) | 22 (66.7) |  |
| 1^st^ degree PEM | 19 (23.5) | 3 (9.1) | 0.12 |
| 2^nd^ degree PEM | 15 (18.5) | 1 (3) | 0.04 |
| 3^rd^ degree PEM | 13 (16.1) | 18 (54.6) | <0.001 |
| **PIM-3 score (%), median (IQR)** | 5.7 (3.3-9.6) | 17.3 (8.5-22.4) | <0.001 |
| **PELOD-2 score, median (IQR)** | 5 (3-6) | 6 (3-7) | 0.03 |

MV, Mechanical Ventilation; PEM, Protein Energy Malnutrition; PIM-3, Pediatric Index of Mortality 3; PELOD-2, Pediatric Logistic Organ Dysfunction 2; Differences in continuous between two groups using a Wilcoxon rank sum test; Differences in categorical variables were used Chi-square test or Fisher exact test

**Supplementary Table 6 |** Treatment information between survivor and death groups.

|  | **Survivor**  **(N=81)** | **Deaths (N=33)** | ***P-*value** |
| --- | --- | --- | --- |
| **Inotropic or vasopressor usage, n (%)** | 41 (50.6) | 26 (78.8) | 0.006 |
| **Maximum VIS in the first 72 hours, median (IQR)** | 2.8 (0-15) | 20 (7-47) | <0.001 |
| **Minimum P/F ratio in the first 72 hours, median (IQR)** | 190 (132-275) | 111 (96.2-165) | <0.001 |
| **Maximum DP (cmH_2_O), median (IQR)** | 15 (12-18) | 19 (16-22) | <0.001 |
| **Muscle relaxant usage, n (%)** | 31 (38.3) | 25 (75.8) | <0.001 |
| **Duration of muscle relaxant (days), median (IQR)** | 3 (2-5) | 10 (5-15) | 0.005 |
| **Cumulative FA Day1 (%), median (IQR)** | 2.8 (0.6-4.4) | 2.6 (0-4.2) | 0.46 |
| **Cumulative FA Day2 (%), median (IQR)** | 4.6 (1.5-7.7) | 1.6 (-1.0-5.2) | 0.01 |
| **Cumulative FA Day3 (%), median (IQR)** | 5.6 (1.4-9.6) | 2 (0.3-7.6) | 0.07 |
| **Cumulative FA Day4 (%), median (IQR)** | 5.6 (0.4-11.1) | 3.9 (0.9-9.3) | 0.30 |
| **Cumulative FA Day5 (%), median (IQR)** | 5.3 (0.4-12.3) | 5.2 (-0.1-9.5) | 0.40 |
| **Cumulative FA Day6 (%), median (IQR)** | 7.4 (-1.0-14.1) | 6.2 (1.4-11) | 0.79 |
| **Cumulative FA Day7 (%), median (IQR)** | 6.5 (-0.5-17.6) | 6.5 (1.4-13.1) | 0.80 |
| **Maximum cumulative FA (%), median (IQR)** | 9.6 (5.5-17.7) | 9 (5.8-18.6) | 0.87 |
| **Days of maximum cumulative FA, median (IQR)** | 6 (3-7) | 6 (3-7) | 0.63 |
| **Respiratory complication during MV, n (%)** |  |  | 0.09 |
| No complication | 15 (18.5) | 2 (6.1) |  |
| Yes | 66 (81.5) | 31 (93.9) |  |
| VAP | 63 (77.8) | 30 (90.9) | 0.12 |
| Pneumothorax | 7 (8.6) | 7 (21.2) | 0.06 |
| Pulmonary hemorrhage | 4 (4.9) | 4 (12.1) | 0.17 |
| Unplanned extubation | 1 (1.2) | 1 (3) | 0.50 |
| Pneumomediastinum | 0 | 0 |  |

PMV, Prolonged Mechanical Ventilation; VIS, Vasoactive-Inotropic Score; DP, Driving Pressure; FA, Fluid Accumulation; Differences in continuous between two groups using a Wilcoxon rank sum test; Differences in categorical variables were used Chi-square test or Fisher exact test

**Supplementary Table 7 |** Logistic regression models assessing the variables associated with deaths.

|  | **Univariate analysis** | | **Multivariate analysis** | |
| --- | --- | --- | --- | --- |
|  | **OR (95% CI)** | ***P-*value** | **aOR (95% CI)** | ***P-*value** |
| **Age < 12 months** | 1.26 (0.56-2.84) | 0.57 |  |  |
| **Female** | 1.46 (0.65-3.31) | 0.36 |  |  |
| **Main diagnosis** |  |  |  |  |
| Pulmonary disease | 0.78 (0.35-1.77) | 0.56 |  |  |
| Cardiac disease | 3 (1.07-8.43) | 0.04 |  |  |
| Neurological disease | 0.8 (0.2-3.16) | 0.75 |  |  |
| Shock | 1.06 (0.26-4.36) | 0.94 |  |  |
| **Underlying disease** | 2.38 (0.74-7.59) | 0.14 |  |  |
| Pulmonary disease | 0.28 (0.06-1.32) | 0.11 |  |  |
| Cardiovascular disease | 3.09 (1.14-8.35) | 0.03 |  |  |
| Gastrointestinal disease | 3.38 (1.11-10.28) | 0.03 |  |  |
| Neuromuscular disease | 0.39 (0.05-3.38) | 0.39 |  |  |
| Oncological disease | 0.81 (0.15-4.22) | 0.80 |  |  |
| Primary immune deficiency | 8 (0.8-79.93) | 0.08 |  |  |
| **Prematurity** | 0.86 (0.34-2.19) | 0.75 |  |  |
| **Respiratory complication** |  |  |  |  |
| VAP | 2.85 (0.78-10.45) | 0.11 |  |  |
| Pneumothorax | 2.84 (0.91-8.89) | 0.07 |  |  |
| Pulmonary hemorrhage | 2.65 (0.62-11.32) | 0.19 |  |  |
| **3^rd^ degree PEM** | 6.28 (2.54-15.54) | <0.001 | 5.14 (1.57-16.88) | 0.01 |
| **Inotropic or vasopressor usage** | 3.62 (1.41-9.29) | 0.01 |  |  |
| **Maximum VIS in the first 72 hours ≥ 14** | 4.69 (1.98-11.11) | <0.001 |  |  |
| **PIM-3 score ≥ 14** | 11.01 (4.3-28.17) | <0.001 | 6.75 (2.26-20.15) | <0.001 |
| **PELOD-2 score ≥ 15** | 1.5 (0.63-3.57) | 0.36 |  |  |
| **Minimum P/F ratio in the first 72 hours < 135** | 3.03 (1.31-7) | 0.01 |  |  |
| **Maximum DP ≥ 17 cmH_2_O** | 3.4 (1.45-7.98) | 0.01 |  |  |
| **Muscle relaxant usage** | 5.04 (2.02-12.57) | 0.001 | 5.58 (1.65-18.86) | 0.01 |

VAP, Ventilation associated pneumonia; PEM, Protein Energy Malnutrition; VIS, Vasoactive-Inotropic Score; PIM-3, Pediatric Index of Mortality 3; PELOD-2, Pediatric Logistic Organ Dysfunction 2; DP, Driving Pressure; OR: Odds ratio, aOR : Adjusted odds ratio , Univariate and multivariate were performed by logistic regression model. Multivariate were developed by covariate with *P* < 0.1 from univariate and step wise backward LR to select final model.
